# Osteoporosis Syndrome in Thalassaemia Major: An Overview

**DOI:** 10.4061/2010/537673

**Published:** 2010-05-26

**Authors:** Meropi Toumba, Nicos Skordis

**Affiliations:** Paediatric Endocrine Unit, Department of Paediatrics, Makarios Hospital, 1474 Nicosia, Cyprus

## Abstract

Osteoporosis in thalassaemia major (TM) represents a prominent cause of morbidity. The mechanism of pathogenesis of bone disease (BD) in TM is multifactorial and complicated. Peak bone mass is achieved shortly after completion of puberty and normally remains stable until the third decade of life when age-related bone mass begins. Growth hormone (GH) and sex steroids play a crucial role in bone remodeling and in the maintenance of skeletal architecture during adult life. GH and insulin growth factors (IGFs) have anabolic effect in bone formation. Sex steroids act probably by increasing the expression of RANKL by osteoblastic cells and alterations in the RANK/RANKL/OPG system in favor of osteoclasts. Impaired GH secretion and lack of sex steroids in thalassemic patients due to pituitary damage, contribute to failure of achieving optimal peak bone mass. Other endocrine complications such as hypoparathyroidism and vitamin D deficiency have also a detrimental role on bones in TM. It is still questionable whether the international criteria for defining osteopenia and osteoporosis are relevant to patients with TM; also a question arises for the diagnostic methods such as DEXA scan and management of osteoporosis with known treatment protocols, in the thalassaemic patient.

## 1. Introduction

Osteoporosis is a universal medical problem, affecting both genders. It is generally accepted that its main causes are aging, genetic disorders of osteogenesis, lack of certain nutritional elements or physical activity, and endocrine disorders mainly estrogen deficiency. Other causes include neoplastic disorders, gastrointestinal disorders causing malabsorption, liver diseases, inflammatory conditions, and drugs. Osteopenia and osteoporosis represent prominent causes of morbidity in patients of both genders with thalassaemia [1]. During the last decade, the presence of osteopenia and osteoporosis in well-treated thalassaemics has been described in different studies with high prevalence up to 50% [2]. The pathogenesis of osteoporosis in thalassaemia major (TM) is complicated and differs from the pathogenesis of bone deformities characteristically found in nontransfused patients who develop bone distortion mainly due to ineffective haemopoiesis and progressive marrow expansion [3]. 

Several factors are implicated in reduction of bone mass in TM. Delayed sexual maturation, growth hormone (GH) and insulin growth factor-(IGF)-1 deficiency, parathyroid gland dysfunction, diabetes, hypothyroidism, ineffective haemopoiesis with progressive marrow expansion, direct iron toxicity on osteoblasts, as well as liver disease have been indicated as possible etiological factors for thalassaemia-induced osteoporosis [2, 3]. Furthermore, iron chelation has correlated with growth failure and bone abnormalities, and high desferrioxamine dosage has been associated with cartilage alterations [2, 4, 5].

## 2. The Role of RANK/RANKL/OPG System

Two distinct cell types are involved in the maintenance and renewal of bone: (A) osteoblasts—cells of mesenchymal lineage responsible for bone formation and (B) osteoclasts—cells of hematopoietic lineage responsible for bone resorption and remodeling. In thalassemia patients, progressive “aging” of the bone starts even in childhood by the gradual development of an imbalance between augmented osteoclastic resorption and insufficient osteoblastic bone formation. 

The bone marrow stromal cell molecules which are members of the tumor necrosis factor (TNF) receptor superfamily, receptor activator of nuclear factor-kappa B ligand (RANKL), receptor activator of nuclear factor-kappa B (RANK), and osteoprotegerin (OPG), play a central role in bone remodeling via conjunction with various cytokines and calciotropic hormones [6]. GH and IGF-1 stimulate the production of OPG and its accumulation in the bone matrix. Sex steroids act probably by increasing the expression of RANKL by osteoblastic cells [6]. Alterations in the RANK/RANKL/OPG system in favor of osteoclasts are characteristic in thalassaemia due to complicated mechanisms involving chronic anemia, iron toxicity, and endocrine complications. 

In patients with Thalassaemia, elevated markers of bone resorption such as serum alkaline phosphatase, osteocalcin, urinary levels of N-telopeptides of collagen type I (NTX), serum levels of tartrate resistant acid phosphatase isoform 5b (TRACP-5b), pyridinoline, and deoxypyridinoline are found. Such elevated markers reveal increased osteoclastic activity and enhanced osteoblastic dysfunction [7–12]. 

Several studies have recently proven that the ratio of sRANKL/OPG is increased in patients with TM and low bone mineral density (BMD), providing evidence for the role of RANKL/OPG system in the pathogenesis of osteoporosis in thalassaemia. The increase of RANKL, followed by unmodified OPG levels, with the consequent increase of RANKL/OPG ratio may represent the cause of uncoupling on bone turnover observed in thalassaemia patients [2, 9, 12]. A negative correlation between the sRANKL/OPG ratio and free testosterone in male thalassaemia patients and between 17-b oestradiol in female thalassaemia, which has also been proven, speculates the role of RANKL/OPG system on the action of sex steroids on bone [12].

## 3. Hypogonadotrophic Hypogonadism

It is well known that sex steroids regulate skeletal maturation and preservation in both men and women. The impact of gonadal insufficiency on skeletal integrity has been widely recognized in adult men and women ever since. Androgens can be converted into estrogens within the gonads and peripheral tissues and both are present in men and women. Sex steroid signaling via sex steroid receptors found in bone possibly affects bone formation and preservation. The exact role of steroids in the pathogenesis of osteopenia is not completely clear. However, it is suggested that sex steroids may act by altering the normal balance of the OPG/RANKL system. Sex steroids increase the expression of RANKL by osteoblastic cells [12]. Oestrogen and progesterone appear to inhibit osteoclastic activity and promote osteoblastic activity [13] while testosterone has a direct stimulatory effect on osteoblast proliferation and differentiation [14]. Thus in the lack of steroids, the osteoclastic activity increases resulting in osteopenia.

Association between hypogonadotrophic hypogonadism and osteoporosis in adult patients with TM has been reported in the past [15, 16]. The contribution of sex steroids in addition to other factors on the development of bone disease in TM has been unequivocally proven [2, 17, 18]. Iron deposition on gonadotrophic cells leads to disruption of gonadotrophin production and consequently leads to delayed puberty and hypogonadotrophic hypogonadism. More than 50% of TM females fail to attend menarche and present with Primary Amenorrhea while Secondary Amenorrhea will invariably develop with time especially in patients poorly compliant to chelation therapy [19–21]. Male patients develop hypogonadotrophic hypogonadism and secondary gonadal failure resulting in low testosterone secretion [22, 23]. Primary gonadal failure may also present due to iron deposition on the testes and ovaries [23, 24]. 

Patients carrying certain genetic defects have an increased degree and rate of iron overloading through transfusions over the years and their Ferritin levels poorly correlate with their total body iron concentrations as proven by the increased frequency of hypogonadism. The role of the genotype as an independent risk factor for the development of endocrine complications in patients with TM on chelation therapy can be explained by the differences in the actual and realistic haemosiderosis, because the patients with the more severe defects have a greater rate of iron loading through higher red cell consumption. [15]. This observation is further supported by the finding of our previous study, which showed that gonadal function was the only factor strongly associated with decreased BMD when the patients were grouped based on genotype [16].

Throughout childhood, BMD normally rises at a steady rate until the age of about 12 years and then there is a sudden acceleration of bone mineral accretion, which coincides with the onset of puberty and the pubertal growth spurt. In patients with thalassaemia, BMD which is already low in childhood [25] decreases further during and after puberty especially in patients with absent or delayed puberty [26]. Bielinski et al. have proved that thalassemic adolescents who failed to progress normally through puberty also failed to preserve adequate bone mineralization and achievement of peak bone mass [27]. Other studies have shown suboptimal bone accrual, regardless normal or induced puberty [1, 28, 29]. 

Reduced bone mass is definitely more common among adult patients with TM due to hypogonadism which has been proposed as a mechanism for osteopenia on this condition. In adult TM patients, the prevalence of osteoporosis and osteopenia is above 50%, and vertebral fractures have been reported in up to 20% of patients [8]. However many other factors including the severity of the hemoglobinopathy, concomitant treatments, and gender differences in bone mass acquisition and maintenance are implicated in bone metabolism in hypogonadal thalassemics. Regarding the hormonal replacement therapy (HRT), there is conflicting evidence on its clinical effectiveness in maximizing bone mass in TM patients. Some studies have shown beneficial effect of HRT especially in younger patients [8, 11] while some others have shown little or no effect of HRT on BMD in thalassaemics who presumably have not achieved peak bone mass [30]. 

## 4. GH-IGF Axis

Although sex steroids are essential for the preservation of BMD during adolescence and adulthood, insulin growth factors (IGFs) and GH play a crucial role on bone formation during childhood and bone maintenance later in life. A number of clinical studies have provided evidence for potential effect of GH/IGF system on bone mass. However, our understanding of the regulation of production and actions of GH/IGF system on bone is incomplete. The anabolic effects of GH and IGF-1 in bone are important for the acquisition of bone mass during adolescence and possibly for the maintenance of skeletal architecture during adult life. The changes in GH and IGF-I secretion that occur with aging are paralleled by a progressive loss of muscle mass and strength, a decline in physical performance, an increase in body fat, and a decrease in BMD [31]. The skeletal effects of GH and IGF are modulated by complex interactions between circulating IGF-I and IGF-binding proteins (IGFBPs) and the locally produced IGF-1 and IGFBPs. 

Growth hormone stimulates the proliferation of cells of the osteoblastic lineage [32] although IGF-1 is required for selected anabolic effects of GH in osteoblasts [33]. Specifically, GH affects the fate of mesenchymal precursors favoring osteoblastogenesis and chondrogenesis and opposing adipogenesis [33]. In addition, it stimulates the expression of bone morphogenetic proteins, which are important for the differentiation of osteoblasts, bone formation [34] and the production of OPG and its accumulation in the bone matrix [35]. Via these mechanisms, GH stimulates longitudinal bone growth, either directly or through an effect mediated by the local IGF-1 [35]. 

The fundamental role of IGF-1 is the stimulation of osteoblastic function and bone formation. IGF-1 has modest effects on the proliferation of cells of the osteoblastic lineage and enhances the function of the mature osteoblast [36]. Additionally, IGF-1 upregulates collagen synthesis and decreases its degradation, which is important for maintaining the appropriate levels of bone matrix and bone mass. Less clear is the function of IGF-1 on osteoclasts. Osteoclasts express IGF-1 receptors and IGF-1 has direct effects on their function [37].

Impaired GH secretion is not a rare occurrence in adult thalassaemics, which contributes to osteopenia and osteoporosis. However, as it was proven in the past the majority of TM patients have the same GH secretion as in the normal population [38]. Some studies have shown impaired GH response to GH-challenge tests in TM patients aged 10–23 years [39, 40]. Others have reported 3.1% of thalassemics to have GHRH-GH-IGF-1 axis dysfunction [41], which is attributed to multiple mechanisms such as neurosecretory dysfunction, hypothalamic GH-releasing hormone (GHRH) deficiency, and increased somatostatin activity [42, 43]. Additionally, thalassemic patients with delayed puberty do not exhibit normal growth spurt; their GH peak amplitude is reduced as well as their nocturnal GH levels [42, 44]. Thalassemics are also proven to have low IGF-1 levels in high prevalence regardless normal or subnormal GH secretion [45]. Recently, it has been reported that defective GH secretion and diminished serum IGF-1 levels may contribute to femoral demineralization in TM patients [45]. In the favor of further investigation, it is valuable that GH status should be retested in thalassaemic patients with childhood onset of GHD [46]. If the diagnosis of adult GHD is established, GH treatment is worth considering as it could contribute to improve bone mineral density. 

There are no data of GH treatment in adults with thalassaemia and how this treatment may contribute to the improvement of BMD of these patients. In a study of Sartorio et al., one year of GH treatment in thalassemic children is able to increase, but not normalize, bone turnover; however it is insufficient to improve BMD values [47]. Prolonged periods of GH therapy are probably requested to positively affect both bone turnover and BMD values in GH deficient thalassaemic patients, as it occurs in children and adults with GH deficiency.

## 5. Hypoparathyroidism and Vitamin D Deficiency

Hypoparathyroidism is another endocrine complication in thalassaemia, which may develop in late adolescence and contribute to osteopenia and subsequently osteoporosis. A recent study has reported prevalence up to 13.5% with no sex differences [48]. Iron overload with deposition on parathyroid cells and tissue fibrosis are the main causes of hypoparathyroidism while chronic anemia is an additional factor causing parathyroid dysfunction [49]. The condition presents with the typical biochemical picture of hypoparathyroidism of low calcium and high phosphate levels. PTH may be normal or low and vitamin D is low. Low calcium and phosphorus are found in 24-hour urine collection. Bone X-rays are characteristic for osteoporosis. Abnormal cerebral CT findings are reported to be related with hypoparathyroidism in thalassemics [48, 49]. 

Vitamin D deficiency may start early in thalassemics, before hypoparathyroidism is established. Vitamin D deficiency potentially contributes to low bone mass in thalassaemia. Notably, TM patients progressively develop iron overload, and it is possible that a deficiency in liver hydroxylation of vitamin D, or in vitamin D absorption, appears in older thalassemic patients. However, studies in children [50, 51] and in adult thalassemic patients [52, 53] have shown contradictory results. Voskaridou et al. evaluating 45 adult TM patients reported that serum vitamin D (25-OH and 1,25-OH-vitamin D) levels were within normal limits in almost all patients [8]. Conversely, Praticò et al. [53] observed that 32 of 113 thalassemic patients (including children and adults) had low serum levels of 25-OH-vitamin D. Other studies have proved a disturbance in the circulating levels of 25-OHD in thalassaemic patients which is aggravated with increasing age [54]. It is difficult to explain these differences due to the multiple factors implicated in the mechanism of vitamin D deficiency. Additionally in countries with poor sunlight, vitamin D deficiency is more common even in the normal population. It is, though, important to consider solar irradiation as a vital factor taking action in the metabolism of vitamin D.

Inaddition to vitamin D, vitamin C and trace elements such as zinc and cooper are involved in the bone metabolism. Vitamin and trace mineral deposition is proven to be inhibited by desferrioxamine in thalassemic patients who receive inappropriately high doses of this chelating agent. Mineral depletion may result in decrease of alkaline phosphatase activity, a zinc-dependant enzyme. Direct toxicity of desferrioxamine by inhibiting cell proliferation, DNA synthesis, and collagen formation cause bone damage resulting in platyspondylosis with flattening of the vertebral bodies and consequently shortening of the spinal height [5]. 

On the other hand iron deposition in bone may impair osteoid maturation and inhibit mineralization locally, resulting in focal osteomalacia. Iron interferes in osteoid maturation and mineralization by the incorporation into crystals of calcium hydroxyapatite which consequently affects the growth of calcium hydroxyapatite crystals and increases osteoid in bone tissue [28]. A damaged bone with small size may simply contribute to the markedly low BMD seen in the thalassemic patients.

## 6. Gender Differences in Bone Disease in TM

**Figure 1:**
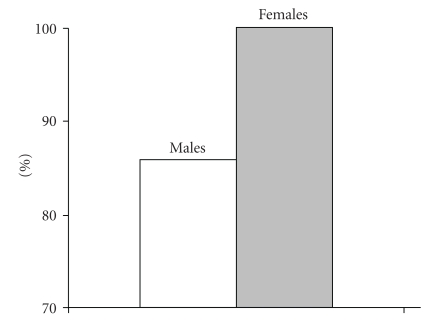
Prevalence (%) of osteoporosis osteopenia syndrome in hypogonadal male and female patients with TM.

**Figure 2:**
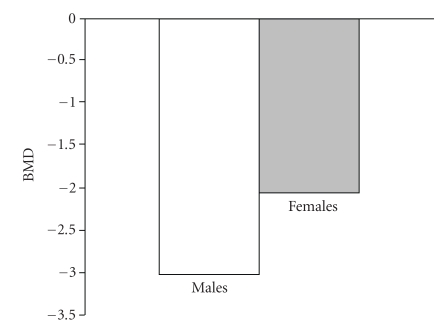
Mean BMD (spine) values in non-hypogonadal male and female patients with TM.

The impact of gonadal insufficiency on skeletal integrity has been widely recognized in both genders. It is though unclear whether there are gender differences in bone disease in TM. Some studies support that there is a gender difference not only in the prevalence but also in the severity of osteoporosis syndrome in TM. A recent study by our institution showed that male patients were more frequently and more severely osteopenic/osteoporotic than females (Figure 1) [55]. Other studies also showed male predominance on osteoporosis although some other reported no gender variation [56–60]. On the other hand, primary amenorrhea and, furthermore, hypogonadism are proven to have a greater impact on osteoporosis in females rather than in males [56]. Additionally, eugonadal females are less severely affected, on spine, compared with eugonadal males most likely due to the beneficial effect of HRT in women with TM (Figure 2) [55]. Alternatively, the bones of male patients are more vulnerable to the adverse effect of other contributing factors, which operate with a complicated and still unknown mechanism. However, some studies showed no gender differences in hypogonadal patients with TM [59, 60].

## 7. Diagnostic Methods of Osteoporosis Syndrome in TM

BMD is generally measured by the Dual Energy X-ray Absorption (DEXA) method. However, it is known that TM patients have spinal degenerative skeletal changes, which can be detected only by MRI and more likely interfere with BMD values, resulting in false diagnosis of bone disease (BD). Therefore, DEXA scans may fail to provide accurate and precise information on osteoporosis in thalassaemic patients. This probably explains the discrepancy between the findings of osteoporosis in TM in different studies. For example in a recent Iranian study by Shamshirsaz et al. the prevalence of osteoporosis was found in 44% of the patients by using the DEXA method whereas only 6% of the same population were osteoporotic based on the Quantitative Computed Tomography (QCT) [59].

One additional contributing factor that interferes with BMD readings in DEXA method is short stature of thalassaemia patients, attributed mostly in the shortening of the spine. The reason is that DEXA measurements are influenced by size and that BMD represents a measurement of bone area rather than volume, leading to underestimation of bone density in such individuals. In summary, since DEXA may fail to provide accurate information of BD in thalassaemia, other methods, such as QCT, high resolution computed tomography and single energy quantitative computed Topography (SEQCT) should be considered as being more sensitive and reliable to detect bone disease in this condition.

## 8. General Aspects in Management of Osteoporosis in TM

The gold key in the management of osteoporosis in patients with TM, in whom, loss of bone mass starts early, is prevention. Treatment of anemia with regular transfusions and the management of iron overload with prompt chelation are mandatory for every patient with thalassaemia in order to avoid adverse events of the disease on bones. Additional lifestyle measures should be encouraged such as physical activity and smoking quitting. Adequate calcium and vitamin D intake during skeletal development can increase bone mass in adult life with the final goal to prevent bone loss and fractures. Early recognition and management of endocrine complications of thalassaemia are essential for eliminating the risk of BD. Induction of puberty at a proper age and treatment of hypogonadism with HRT seem to be the most effective ways for preventing osteoporosis and other bone deformities in thalassaemia [17]. Calcitonin, a potent inhibitor of osteoclasts, has also been tried in combination with calcium and vitamin D [60]. Bisphosphonates are worldwide used in patients with postmenopausal osteoporosis to increase BMD and prevent bone fractures [61]. The reduction in fractures may be related not only to the increase in bone mass arising from the inhibition of bone resorption and reduced activation frequency of bone-remodeling units but also to enhanced osteomineralization. Alendronate, pamidronate, and zoledronic acid seem to have greater efficacy in osteoporotic patients with TM either with normal or impaired gonadal function [8, 9, 62, 63]. Since the origin of bone disease in TM is multifactorial and some of the underlying pathogenic mechanisms are still unclear, further research in the therapeutic trials with bisphosphonates is needed to allow definite conclusions.

## 9. Conclusion

It is obvious that osteoporosis in thalassemia is of multifactorial etiology and of complex mechanism which need to be clarified. Endocrine complications mentioned in this paper inaddition to progressive marrow expansion, iron and desferrioxamine toxicity on bones, as well as liver disease contribute to the complex mechanism of osteoporosis in thalassaemia patients. Thus, osteoporosis in TM represents a unique clinical entity, which adversely affects the life expectancy of patients with TM. It is still questionable whether the international criteria for defining osteopenia and osteoporosis are relevant to patients with TM; also a question arises for the diagnostic methods and management of osteoporosis in the thalassaemic patient as multiple factors are involved in its pathogenesis. Close follow-up early recognition of osteopenia, as well as proper management are crucial for every thalassemic patient giving him the right to live a better life.
